# Complete Genome Sequence of *Tepidibacter* sp. Strain 8C15b, Isolated from Bank Sediments of Haiphong Bay, Vietnam

**DOI:** 10.1128/mra.00132-22

**Published:** 2022-06-27

**Authors:** Stéphanie Fouteau, Vuong Bui Van, Zoé Rouy, Mélanie Beraud, Corinne Cruaud, Jean-Luc Cayol, Sandrine Chifflet, Thuoc Chu Van, Thu Pham The, David Vallenet, Pedro H. Oliveira, Xavier Mari, Nathalie Pradel

**Affiliations:** a Génomique Métabolique, CEA, Genoscope, Institut de Biologie François Jacob, Université d’Évry, Université Paris-Saclay, CNRS, Évry, France; b Institute of Marine Environment and Resources (IMER-VAST), Haiphong, Vietnam; c Genoscope, Institut de Biologie François-Jacob, Commissariat à l'Energie Atomique (CEA), Université Paris-Saclay, Évry, France; d Aix Marseille Université, Université de Toulon, CNRS, IRD, MIO, Marseille, France; e University of Science and Technology of Hanoi (USTH-VAST), Hanoi, Vietnam; Montana State University

## Abstract

We report the complete genome sequence of *Tepidibacter* sp. strain 8C15b, isolated from bank sediments of Haiphong Bay, Vietnam. The genome includes a 3,628,320-bp circular chromosome and a plasmid of 38,213 bp.

## ANNOUNCEMENT

Haiphong is the second largest harbor in Vietnam, subject to high levels of anthropogenic disturbance (1). We isolated and sequenced the genome of a strain designated *Tepidibacter* sp. strain 8C15b, from estuarine sediments of Haiphong Bay (20°45′28.39″N, 106°46′22.91″E). The sediments were collected using a piston corer. Enrichment and isolation of strain 8C15b were performed from 200 mg of sediment using the Hungate technique (2). For isolation, the culture was serially diluted in roll tubes until the isolate was axenic (3). DSMZ medium 985 was used to grow the strain at 30°C. DNA was extracted from 5 mL of pure culture using the Wizard genomic DNA purification kit (Promega).

Genome sequencing was carried out using a combination of Illumina and Oxford Nanopore Technologies. For Illumina sequencing, 250 ng DNA was sonicated to the 100 to 1,000-bp size range using the E220 focused ultrasonicator (Covaris, Inc.). The fragments were end repaired and 3′ adenylated, and NEXTflex HT barcodes were added (BioScientific Inc.). The ligated product was amplified using 12 PCR cycles with the Hifi HotStart next-generation sequencing (NGS) kit (Kapa Biosystems) and purified with 0.6× AMPure XP reagent. After library profile analysis using the Agilent 2100 Bioanalyzer and quantitative PCR (qPCR) (MxPro; Agilent Technologies), the library was sequenced on an Illumina MiSeq instrument with a MiSeq reagent kit v.2 (2 × 250 bp). A total of 2.27 × 10^6^ paired-end reads were obtained. The Illumina reads were trimmed by removing low-quality (Q < 20) nucleotides, sequencing adaptors, and sequences of <30 nucleotides (nt), using the FastX-Toolkit package. For Nanopore sequencing, library preparation was conducted using 800 ng of the same input DNA following the 1D genomic DNA protocol with the SQK-LSK110 ligation kit (Oxford Nanopore). The library was sequenced using a Nanopore R9.4.1 revD flow cell and the GridION device with MinKNOW v.4.3.4 and Guppy v.5.0.11 + 2b6dbff software. A total of 338,403 reads were obtained with an *N*_50_ value of 2.66 kb. Two hybrid assemblies were launched using Unicycler v.0.4.6 (one with default settings and one with the –sc option) and compared using Mummer v.3.23, to resolve the integrative and free circular copies of the plasmid. Overlapping sequences at the contig ends (obtained using the SPAdes process) were removed so that a circular contig was obtained. Default parameters were used for all software unless otherwise specified. The annotation was performed using the Microscope platform (4), and taxonomy analysis was conducted using GTDB-Tk v.1.3.0. The completion and contamination percentages were calculated using CheckM v.1.0.11.

The whole genome consists of a 3,628,320-bp circular chromosome, including an integrative plasmid of 38,213 bp, and a second free circular copy of the plasmid. The average G+C contents of the chromosome and plasmid were 29.79% and 27.28%, respectively. A total of 3,516 coding DNA sequences (CDSs) were predicted for the chromosome and 36 CDSs for the plasmid. NCBI blastn analysis indicated 99% identity over 95% of the length of the 16S rRNA gene sequences of Tepidibacter mesophilus JCM16806, isolated from soil polluted by crude oil in China (5). The average nucleotide identity value between the two genomes was 89.5%. The genome sequence of strain 8C15b may provide a resource for better understanding the mechanisms of adaptation to anthropogenically disturbed environments.

### Data availability.

This whole-genome project and the raw data have been deposited at ENA under the accession number PRJEB50689. The chromosome and plasmid sequences are available at ENA under the accession numbers OW235315 and OW235316, respectively.
